# Differential Impacts of Willow and Mineral Fertilizer on Bacterial Communities and Biodegradation in Diesel Fuel Oil-Contaminated Soil

**DOI:** 10.3389/fmicb.2016.00837

**Published:** 2016-06-02

**Authors:** Mary-Cathrine Leewis, Ondrej Uhlik, Serena Fraraccio, Kelly McFarlin, Anastasia Kottara, Catherine Glover, Tomas Macek, Mary Beth Leigh

**Affiliations:** ^1^Institute of Arctic Biology, University of Alaska Fairbanks, FairbanksAK, USA; ^2^Department of Biochemistry and Microbiology, Faculty of Food and Biochemical Technology, University of Chemistry and TechnologyPrague, Czech Republic

**Keywords:** stable isotope probing, bioremediation, phytoremediation, *Salix alaxensis*, fertilizer, microbial community structure, naphthalene degradation, diesel range organics

## Abstract

Despite decades of research there is limited understanding of how vegetation impacts the ability of microbial communities to process organic contaminants in soil. Using a combination of traditional and molecular assays, we examined how phytoremediation with willow and/or fertilization affected the microbial community present and active in the transformation of diesel contaminants. In a pot study, willow had a significant role in structuring the total bacterial community and resulted in significant decreases in diesel range organics (DRO). However, stable isotope probing (SIP) indicated that fertilizer drove the differences seen in community structure and function. Finally, analysis of the total variance in both pot and SIP experiments indicated an interactive effect between willow and fertilizer on the bacterial communities. This study clearly demonstrates that a willow native to Alaska accelerates DRO degradation, and together with fertilizer, increases aromatic degradation by shifting microbial community structure and the identity of active naphthalene degraders.

## Introduction

Release of diesel and other petroleum hydrocarbons into the environment is a widespread, global problem and can be caused by leaking storage tanks, land disposal of petroleum waste and accidental spills (Bossert and Bartha, 1984). Diesel fuels, used in internal combustion engines, predominantly contain a mixture of hydrocarbons that typically include between 8 and 28 carbon atoms per molecule. These hydrocarbons are mainly composed of alkanes (40–70 mass %), cycloalkanes (10–25 mass %), alkenes (up to 5%) and aromatics (10–30%; Trapp et al., 2001). Consequently, petroleum hydrocarbons are a common environmental contaminant and are of concern because many are chemical carcinogens and can cause chronic toxic effects (Agency for Toxic Substances and Disease Registry, 1999).

Phytoremediation, the use of plants to stimulate environmental degradation of contaminated soils, is a potentially effective alternative for treatment of petroleum hydrocarbon-contaminated soils at lower costs than traditional physical–chemical remediation strategies such as removal and incineration. Costs associated with phytoremediation have been estimated near $162 per m^3^ of petroleum-contaminated soil compared to more than $810 per m^3^ for excavation and incineration (Rock and Sayre, 1998). For hydrophobic petroleum components such as diesel range organics (DRO), rhizoremediation, or phytoremediation in the root zone of plants, is the primary mechanism of degradation (Siciliano et al., 2003; Yi and Crowley, 2007). The presence of roots alters the microbial community in the rhizosphere by increasing microbial density and altering community structure relative to bulk soil (Neumann et al., 2009; Uhlík et al., 2009; Dennis et al., 2010; Yergeau et al., 2014). As roots grow and die back due to fluctuating water tables, soil moisture, and seasonal fine root turnover, they release nutrients and organic carbon, including secondary metabolites, into the surrounding soil (Leigh et al., 2002; Yi and Crowley, 2007; Toussaint et al., 2012). Because plant species differ substantially in their chemical composition, particularly secondary compounds, different plants can foster different rhizosphere communities (Bryant et al., 1992; Agrawal and Fishbein, 2006; Badri et al., 2009). Therefore, some plants may be more effective than others at facilitating microbial biodegradation rates of certain contaminants, particularly in the rhizosphere. However, the mechanism by which plants stimulate rhizodegradation of petroleum products has not yet been fully determined.

Willows are a promising group of woody plants for phytoremediation that have been previously found to stimulate petroleum biodegradation (Leewis et al., 2013; Yergeau et al., 2014; Pagé et al., 2015). The genus *Salix* is a large, taxonomically complex genus, with 300–500 different species found primarily in cold and temperate regions of the Northern Hemisphere (Argus, 1999). Willows are known for their rapid growth, flood tolerance, and ease of vegetative propagation, and their tissues characteristically contain a diverse assortment of phenolics (Julkunentiitto, 1989; Pulford and Watson, 2003), some of which are released in response to wounding or defense (Fields and Orians, 2006; Nováková et al., 2014; Tanaka and Nakamura, 2015). Willows also release phenolic compounds into the rhizosphere through exudation and cell lysis during fall root turnover. The quantity of phenolics released by plants has been shown to increase with age and environmental stresses associated with water, light, oxygen, temperature and nutrient availability (Gaffney et al., 1993; Dakora and Phillips, 2002; Fields and Orians, 2006). In general, willows are considered as a natural source of salicylate, an intermediate in the bacterial naphthalene dioxygenase pathway and inducer of genes encoding for catabolic enzymes of naphthalene and higher-molecular weight polycyclic aromatic hydrocarbons (PAHs; Schell, 1985; Chen and Aitken, 1999; Pagé et al., 2015). Willows are diverse, abundant, and grow robustly in interior Alaska, and although the roots of the native Alaskan *Salix alaxensis* have been shown to stimulate microbial biodegradation of PCBs in microcosm studies (Slater et al., 2011), they have not yet been studied for their ability to rhizodegrade DRO.

We examined the ability of *S. alaxensis* and/or fertilizer to stimulate diesel fuel oil biodegradation in soil, and subsequently applied stable isotope probing (SIP) methods to determine how willows and nutrients affect community structure and the identity of active naphthalene-degrading bacteria. We hypothesized that the identity of naphthalene degraders would differ in rhizosphere compared to bulk soils because of the selective rhizosphere effect, and because the quantity of phenolics released by plants is associated with nutrient availability (Dakora and Phillips, 2002), we hypothesized that willow and mineral fertilizer would have differential and combined impacts on the diversity and activity of bacterial communities in diesel fuel-contaminated soil, including taxa involved in naphthalene biodegradation.

## Materials and Methods

### Soil and Plant Material

Soils were obtained from Great Northwest Inc. (Fairbanks, AK, USA) and were a local blend of 60% silt and 40% peat. Absence of petroleum contamination in soils was verified by GC-MS analysis using a Carlo-Erba C/N analyzer; nitrogen (0.17% dry weight), carbon (2.87% dry weight). Initial values of percent moisture (9.52%) were measured gravimetrically. Initial percentage of organic matter (6.50%) was measured following the ASTM D 2974 dry ashing method. Soil was then spiked with diesel fuel oil #2 (University of Alaska Fairbanks incinerator) to a concentration of DRO 12.96 ± 3.03 mg⋅kg^-1^ by spraying and homogenizing in small batches using a cement mixer.

Willow (*S. alaxensis*) clippings were obtained from a single stand adjacent to the Fairbanks International Airport (Fairbanks, AK, USA; 64°47′40″N, 147°53′45″W) and were rooted vegetatively. Stem cuttings (∼20 cm) were rooted by planting in sterile saturated sand in 100% humidity tents in the University of Alaska Fairbanks Greenhouse. Cuttings were saturated three times a week with a water-fertilizer solution (52 mg⋅L^-1^ Ca, 3.4 mg⋅L^-1^ Mg, 145 mg⋅L^-1^ total N, 76 mg⋅L^-1^ P and 125 mg⋅L^-1^ K) to promote root growth. After 2 weeks, clippings had sufficient root growth to sustain the newly formed leaves and were subsequently acclimated to outside conditions prior to planting into the diesel-contaminated soil. At the time of planting, the clippings were carefully removed from the sand, rinsed, and transplanted into pots containing diesel-contaminated soil.

### Pot Incubations

One-liter plastic pots were filled with diesel-contaminated soil and three grab samples (∼50 g each) were taken as the soil was placed in each pot: one when the soil filled 1/3 of the pot, another when the soil filled 2/3 of the pot’s volume, and the final sample when the soil was approximately 2.5 cm from the top of the pot. The three grab samples from each pot were then combined, homogenized and sampled for later DRO quantification and most probable number (MPN) analyses of diesel-degrading microorganisms (DDM).

Pots were randomly assigned to one of the four treatments: bulk soil (unplanted, unfertilized, labeled *B*), fertilized bulk soil (*BF*), willow-vegetated soil (*W*), and willow-vegetated fertilized soil (*WF*), each with nine replicates per treatment. Pots were randomly placed in an outdoor grid with pots set in plastic bins (2–3 pots per bin) in order to ensure that drainage from fertilized treatments was separated from non-fertilized treatments. All bins were placed in a wooden frame lined with plastic to contain any contaminated runoff in the event of heavy rain and the surface of the pots were covered with shade cloth to help maintain natural low-temperature soil conditions. All pots were watered on the first day and then as needed for the remainder of the experiment. Fertilized treatments were treated with 500 mL watering of the following composition: 100 mg⋅L^-1^ Ca, 6.8 mg⋅L^-1^ Mg, 290 mg⋅L^-1^ total N, 153 mg⋅L^-1^ P, and 250 mg⋅L^-1^ K two times per week for the total duration of the experiment. The experiment was run for 3 months.

At the time of destructive harvesting, plants were separated into roots, stems, and leaves for biomass determinations. The stem was cut at the soil surface, any attached leaves were removed, coarse roots (>1.0 mm) were carefully separated from the soil, and the remaining soil was homogenized and sampled for DRO concentration, MPN analysis and SIP microcosm construction. Roots (coarse > 1.0 mm and fine < 0.075 mm) and shoots were washed. Collected plant biomass was dried for 48 h at 60°C and weighed to determine dry weight.

### DRO Concentration Analysis

DRO concentrations, defined as the sum of alkanes and aromatics from C_10_ to C_25_, were determined by GC/MS analysis. The extraction procedure was adapted from Schwab et al. (1999). Briefly, samples were thawed for 2 h at room temperature before three replicates of 1.00 ± 0.03 g of contaminated soil were added to clean 15-mL glass centrifuge tubes. Using a glass syringe, 250 μL of 893 mg⋅L^-1^ naphthalene-d8 (99 atom % D, Sigma–Aldrich, USA) in dichloromethane was added to each sample as a surrogate and allowed to adsorb to the soil particles for 30 min. Ten milliliters of dichloromethane was added to each soil sample and the centrifuge tubes were sealed with a Teflon-lined lid and shaken horizontally on a rotary platform shaker for 30 min (120 rpm). Tubes were then centrifuged at 45,000 × *g* for 10 min and the extract solution decanted. Ten more milliliters of fresh dichloromethane was then added to each tube and the process was repeated for a total of three times, producing 30 mL of extract in total. All extracts were stored at 4°C until DRO analysis in GC vials with Teflon lined septa. Prior to GC analysis, 1 mL of each extract was transferred to a GC vial and spiked with 5 μL of 2,000 ppm 5α-androstane as an internal standard. An internal standard was also added to each calibration standard. Diesel fuel oil #2 was used to prepare stock calibration standards in dichloromethane. Four diesel standards (100 mg⋅L^-1^, 300 mg⋅L^-1^, 600 mg⋅L^-1^, and 1000 mg⋅L^-1^) were run with each sample series. GC/MS analyses were performed using an Agilent 6890N GC with an Agilent 5973 mass selective detector (MS) and a column of 30 m × 320 μm × 0.25 μm. The GC was programmed to run at an initial temperature of 35°C, held for 1 min, then ramped up by 12°C per min to 320°C and held for 15 min. The total run time was 40.75 min. The injection port and the MS detector were both maintained at 280°C using a 1-μL pulsed splitless injection. Peak areas were manually integrated using data output from Agilent MSD Chemstation E02.00. We determined statistical differences in DRO loss by treatment and time using a two-factor ANOVA on log-transformed DRO. Differences between initial and final sampling points were further evaluated using the Mann–Whitney *U* test for non-normal data.

### Enumeration of DDM

The abundance of DDM was determined by a 96-well plate MPN method adapted from Haines et al. (1996). Triplicate 1-g grab soil subsamples were taken from each pot. Microorganisms were extracted by suspending 1 g of soil in 10 mL 1% w/v sodium pyrophosphate (Fisher Scientific, USA) solution with the addition of 3–4 g of glass beads and shaking horizontally on a rotary platform shaker for 1 h. The soil suspension was allowed to settle in upright tubes for 30 min before inoculating MPN plates.

A 96-well plate was set up with 180 μL of sterile Bushnell–Haas (BD, Sparks) medium (Bushnell and Haas, 1941) per well. A 20-μL sample of microbial suspension was pipetted into the first column on the 96-well plate, thoroughly mixed and a subsample of 20 μL was inoculated into the adjacent well, resulting in 10-fold serial dilutions of the sample across the plate. Each plate included three replicate series from each soil suspension, a positive control (microbial suspension, diesel fuel and medium) and two negative controls (medium and medium with diesel fuel, both with no inoculum). After inoculation, 20 μL of filter-sterilized (0.22 μm) diesel fuel oil #2 was added to each well, except the one negative control. The plates were sealed with Parafilm and placed in plastic bags to reduce evaporative loss. All plates were incubated in the dark at room temperature for 14 days. After the 14-day incubation, 50 μL of 3 g⋅L^-1^ *p*-iodonitrotetrazolium (MP Biomedicals, Salon) was filter-sterilized (0.22 μm) and then added to each well. This indicator dye deposits a red precipitate in the presence of active respiring microorganisms. After 48 h the wells were scored, with a red or pink color producing a positive result. The MPN values were calculated from previously published MPN tables (de Man, 1983) and statistically evaluated by an ANOVA followed by a Tukey’s pair-wise comparison after log-transformation of the data to attain normality using the PAST program. Normality of the data set was tested using the Shapiro–Wilk test.

### SIP Microcosms

Triplicate (labeled I, II, III) SIP microcosms were established in 100-mL serum bottles (Sigma–Aldrich, USA). First, 10 μL of a 50 mg⋅mL^-1^ ^13^C-naphthalene (>99% ^13^C, Sigma–Aldrich, USA) solution in acetone was pipetted on the inner wall of the vial, leaving the solvent completely evaporate in a fume hood. Second, 3-g grab samples of soil from each of the pot-study treatments were added and moistened with 500 μL of molecular biology grade water (Sigma–Aldrich, USA). Each vial was then sealed with crimped silicon septa. Each set of triplicate microcosms was destructively harvested after 3 and 7 days (sampling times T1 and T2, respectively) of incubation at laboratory temperature (∼25°C) in the dark, and stored at -80°C until DNA isolation along with samples that were frozen at the time of microcosm set up (referred to as T0).

### ^13^C-DNA Isolation

Total (metagenomic) soil DNA from the samples T0, T1, and T2 was isolated with FastDNA Spin Kit for Soil (MP Bio, USA), according to manufacturer’s instructions. Isolated DNA was quantified with a NanoPhotometer^®^ P-Class (Implen, Germany). All DNA solutions were adjusted to a concentration of 100 ng⋅μL^-1^ and a volume of 8 μL (∼800 ng DNA) of each sample was mixed with 1.6 g⋅mL^-1^ cesium trifluoroacetate (CsTFA) solution (GE Healthcare Life Sciences, UK) in a 2 mL Sorvall centrifuge cuvette (Thermo Scientific, USA). The cuvettes were sealed and subjected to isopycnic ultracentrifugation at 145,000 × *g* for 72 h on a Discovery 90 Ultracentrifuge with a TFT-80.2 Fixed-Angle Ultraspeed Centrifuge Rotor (Sorvall, USA). The isopycnic CsTFA gradient formed was fractionated into thirty fractions by using a Beckman Fraction Recovery System (Beckman Coulter, USA) and Harvard Pump 11 Plus Single Syringe (Harvard Apparatus, USA) with a flow rate of 200 μL⋅min^-1^. The buoyant density of each gradient-recovered-fraction was inferred by measuring the refractive index of a fractionated blank sample, where 8 μL of water were loaded instead of sample, determined with a Digital Handheld Refractometer (Reichert Analytical Instruments, USA). DNA of the gradient fractions with buoyant densities ranging between 1.46 and 1.68 g⋅mL^-1^ was retrieved by isopropanol precipitation with glycogen (Uhlík et al., 2009).

DNA distribution across the selected gradient fractions was assessed by real-time qPCR analysis of the 16S rRNA genes as described by Uhlík et al. (2012). Comparison of the DNA distribution between T0, T1, and T2 defined the range of BD where ^13^C-labeled DNA occurred. These fractions were compiled and further analyzed along with equivalent fractions from T0 samples.

### 16S rRNA Gene Amplicon Pyrosequencing

The preparation of PCR products for pyrosequencing was based on a procedure modified from that described by Berry et al. (2011). Regions V4–V6 of the 16S rRNA gene were amplified with primers 515–530 forward, 5′-GTGCCAGCMGCNGCGG-3′, and 1068–1052 reverse, 5′-CTGRCGRCRRCCATGCA-3′. The PCR reaction was prepared in a final volume of 15 μL with: (i) KAPA HiFi HotStart ReadyMix (Kapa Biosystems, Boston, MA, USA), containing 0.02 U/μL of KAPA HiFi HotStart DNA Polymerase, 2.5 mM MgCl_2_ and 0.3 mM of each dNTP; (ii) 0.3 μM of each primer (Generi Biotech, Czech Republic); (iii) template DNA (1 μL). The cycling program was set as follows: 5 min at 95°C, 35 cycles of 20 s at 98°C, 15 s at 50°C, 15 s at 72°C and a final extension of 5 min at 72°C. Five microliters of the resultant PCR product were utilized as template for a reconditioning PCR, performed in a final reaction volume of 25 μL with 1 μM of each primer. The reconditioning PCR was conducted with the same reagents and concentrations as mentioned above and the same amplification conditions, except that the cycle number was decreased to 12. The forward and reverse primers utilized for the reconditioning PCR were modified with sequencing adapters (454 Sequencing Application Brief No. 001-2009, Roche). The forward primer also contained a barcode sequence (454 Sequencing Technical Bulletin No. 005-2009, Roche) unique for each sample, which allowed for multiplexing. The resultant PCR products were purified using AMPure XP Beads (Agencourt, Beckman Coulter, USA) and the DNA concentration of the purified DNA solutions was measured with Qubit^®^ dsDNA HS (High Sensitivity) Assay Kit (Life Technologies). Proportional amounts of each amplicon were mixed and then unidirectionally sequenced from the forward primer using the GS FLX Titanium chemistry followed by signal processing (Roche) at the Institute of Molecular Genetics, Czech Academy of Sciences, Prague, Czech Republic.

### Pyrosequencing Data Analysis

Pyrosequencing data were processed with the aid of the mothur software package, version 1.31.1 (Schloss et al., 2009) as described previously (Wald et al., 2015). Operational taxonomic units (OTUs) were defined at 3% dissimilarity. Validation of OTUs detected in ^13^C-DNA was performed as follows: if the mean number plus standard deviation of OTU sequences in triplicate T0 samples was higher than the mean number of OTU sequences in triplicate ^13^C-DNA samples, the OTU was considered as non-labeled background contamination of ^13^C-DNA and was excluded from further analyses. Non-metric multidimensional scaling ordination analysis (NMDS) with vector fitting was performed in the *vegan* package (Oksanen et al., 2013) in *R* (R Development Core Team, 2009). Major OTUs responsible for the differences in the communities were identified with mothur-implemented version of metastats (White et al., 2009) and depicted in a heatmap, which was constructed in *R* using *heatmap2* function with a dendrogram created based on Bray–Curtis dissimilarity matrix and average linkage hierarchical clustering. PERMANOVA was performed using the *adonis* function in R to statistically evaluate the significance of treatment on the microbial communities (Anderson, 2001).

### Sequence Deposition

Pyrosequencing reads were deposited in NCBI Short Read Archive under bioproject number PRJNA317613.

## Results

### Changes in Plant Biomass

The addition of fertilizer to pots containing *S. alaxensis* resulted in increased root and stem biomass. A portion of the difference in leaf biomass is due to the timing of harvest, as plants were undergoing different stages of senescence. The majority of the leaves on the unfertilized plants had already fallen at the end of the experiment, while at the same time point, the fertilized plants were at a much earlier stage of senescence, exhibiting small amounts (≤30%) of color change. Aside from the biomass differences accountable to early leaf senescence in the unfertilized plants, the increased root and stem biomass of the fertilized willow treatment indicated that fertilizer contributed to an increase of plant biomass in the investigated soils.

### DRO Loss in Pot Experiments

**FIGURE 1:**
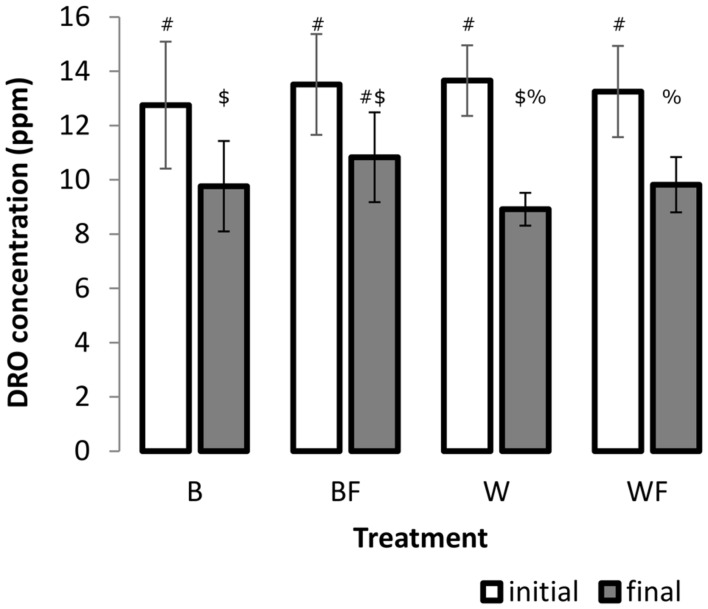
**Concentration of DRO in soil samples before and after the 12-week pot study determined by GC-MS.** Three 1 g soil samples were taken from each pot. A minimum of four analytical standards was analyzed with every run. Error bars represent 95% confidence intervals. Different symbols within the graph refer to significantly different averages based upon the Mann–Whitney *U* test.

A two-factor ANOVA was found significant for log-transformed DRO concentrations across all treatments over the incubation period (*F* = 45.85, *P* ≤ 0.001, **Figure 1**), however, overall there was no significant treatment or interaction effects. We further explored these differences over the incubation period and found that growth of *S. alaxensis* in diesel-contaminated soils resulted in a significantly greater loss of DRO across the 3-month time series than in soils without willows (*P* ≤ 0.001, **Figure 1**). Concentrations of DRO also decreased significantly over time in untreated soils, but to a lesser extent than in willow-treated soils (*P* ≤ 0.05, **Figure 1**). In the treatments with only fertilizer, DRO at the end of the 3-month incubation period was not significantly lower than initial measurements (*P* > 0.05, **Figure 1**).

### Treatment Effects on Community Structure and Quantity of DDM

**FIGURE 2:**
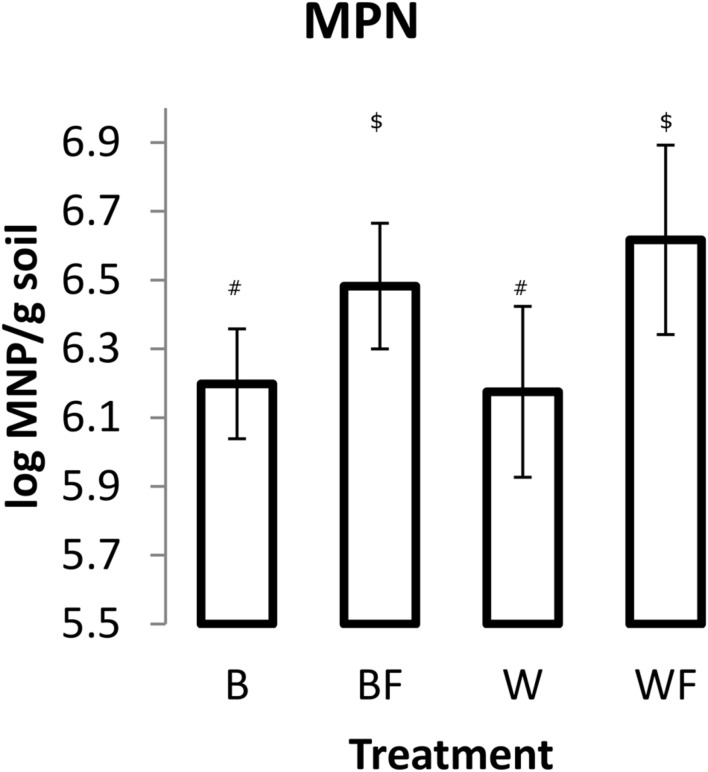
**Most probable number (MPN) of DDM in soils following the 96-day pot study.** Measurements from each pot were based on 10 triplicate 1 g soil samples. The values shown are means with 95% confidence intervals. The results of an unpaired *t*-test showed that treatments BF and WF were significantly different from B (*P* < 0.05). Different symbols within the graph refer to significantly different averages based upon the Mann–Whitney *U* test.

Impact of Alaskan willow and mineral fertilizer on soil microbial communities was assessed through MPN of DDM and pyrosequencing analysis of 16S rRNA genes. MPN data (**Figure 2**) showed that fertilization, both with and without willow plants, resulted in significantly higher numbers (*P* ≤ 0.05) of DDM in comparison to unfertilized soil. The highest mean quantity of DDM was observed in the fertilized willow treatment.

**FIGURE 3:**
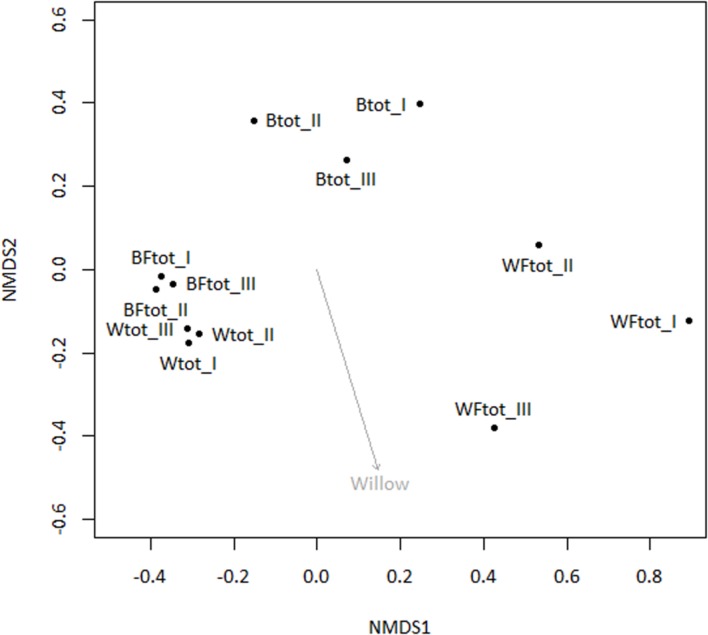
**Non-metric multidimensional scaling ordination analysis (NMDS) of total soil bacterial community (16S rRNA genes) with subsequent fitting of environmental vectors of treatment (willow, fertilizer) onto the ordination (*P* ≤ 0.05)**.

**FIGURE 4:**
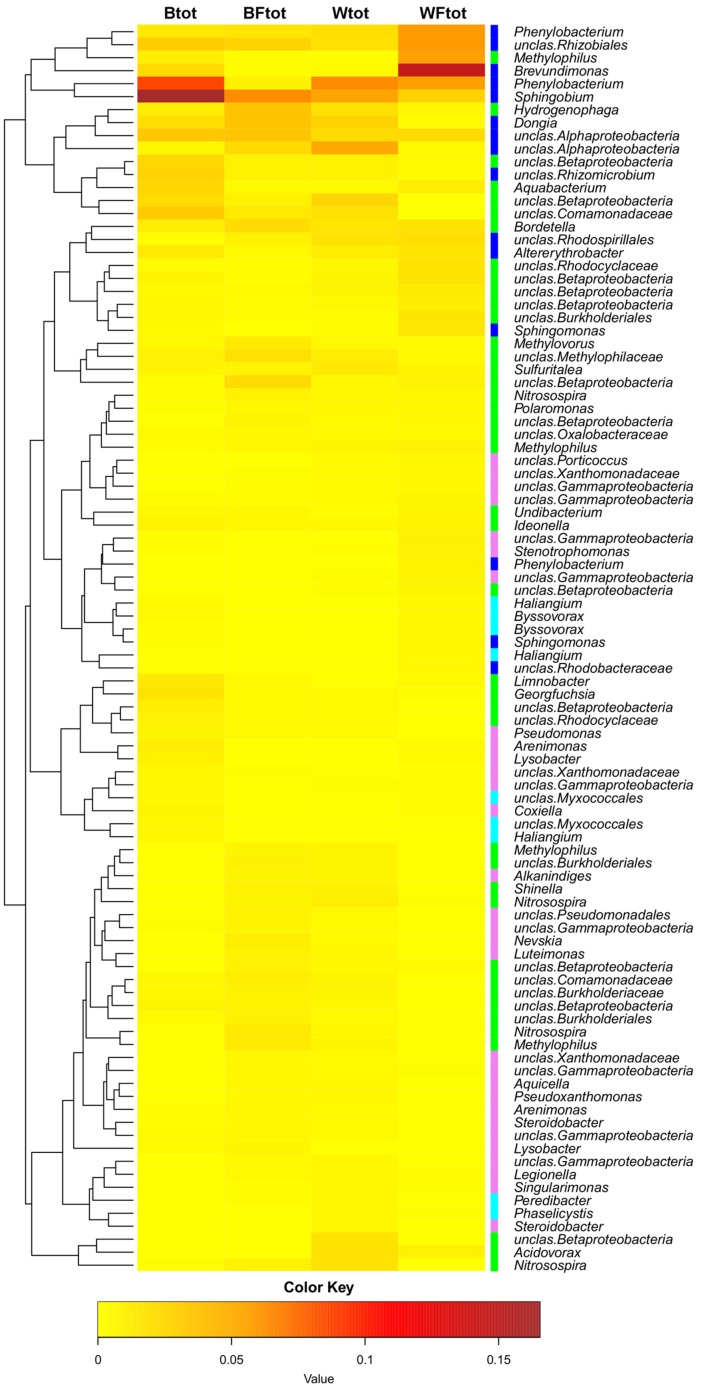
**Heatmap showing the OTUs from the total soil bacterial community that are responsible for differences seen between samples (as determined by metastats).** Only OTUs with abundance ≥0.5% at least in one total community sample are shown. The colors ranging from yellow to brown indicate the gradient in abundance of the OTU. Colors affiliated with the taxa represent the following classes: *Alphaproteobacteria* in blue, *Betaproteobacteria* in green, *Gammaproteobacteria* in pink, *Deltaproteobacteria* in cyan. The dendrogram shows which OTUs occur more often together.

When the entire bacterial community was evaluated using 16S rRNA gene sequencing, NMDS showed clustering of the total soil community based on treatment (**Figure 3**). A vector-fitting method used to interpret the ordination using treatment data indicated that only willow, and not fertilizer, was statistically significant (*P* ≤ 0.05) at explaining the ordination (**Figure 3**). PERMANOVA analyses for the effect of the different treatments on overall bacterial community composition indicated significant results for willow (*F* = 4.40, *P* = 0.01) and fertilizer (*F* = 3.43, *P* = 0.03), and for the interaction between willow and fertilizer (*F* = 8.92, *P* = 0.01). We further analyzed those OTUs that were significantly different (*P* ≤ 0.05) in at least one treatment and were also at least 0.5% abundant in more than one community. This analysis, performed with the mothur-implemented version of metastats (White et al., 2009), indicated that taxa mostly belonging to *Proteobacteria* (from *Alpha*- to *Deltaproteobacteria*) were those which differed in abundance between treatments. Many of these taxa were unclassified at the level of genus, suggesting they were novel sequences of yet-to-be characterized bacteria (**Figure 4**).

### Mineralization of Naphthalene

**FIGURE 5:**
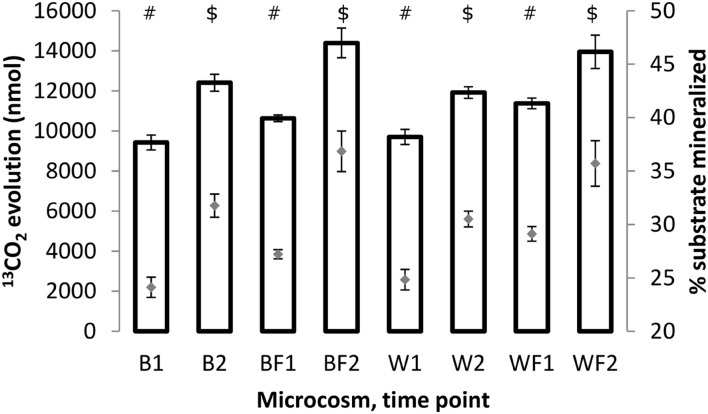
**Amounts of ^13^CO_2_ (bars, left axis) evolved after 3 and 7 days of incubation of the soil samples with ^13^C-naphthalene and percentage amounts of original substrate which has been converted to ^13^CO_2_ (gray diamonds, right axis).** All values shown are averages from biological triplicates, error bars represent standard deviations. The values were calculated based on chemical equation describing naphthalene mineralization: C_10_H_8_ + 12 O_2_ → 10 CO_2_ + 4 H_2_O. Different symbols within the graph refer to significantly different means as tested by an unpaired *t*-test.

Mineralization rates of naphthalene for different treatments were assessed through ^13^CO_2_ quantification in the microcosm headspace. Results (**Figure 5**) showed that the amounts of evolved ^13^CO_2_ (and corresponding amounts of the substrate respired) increased between 3 and 7 days in all treatments. This increase was statistically significant (unpaired *t*-test of means, *P* ≤ 0.01) in all cases. Similarly, the rates of evolved ^13^CO_2_ were significantly higher (unpaired *t*-test of means, *P* ≤ 0.05) for fertilized treatments than non-fertilized ones, indicating a stimulatory effect of fertilization on metabolic potential of microorganisms responsible for naphthalene mineralization.

### Taxa Deriving Carbon from Naphthalene

Taxa deriving carbon either directly from naphthalene or indirectly from naphthalene metabolites and/or dead labeled biomass were identified by SIP coupled to pyrosequencing analysis of 16S rRNA gene amplicons. The qPCR analysis of SIP density gradient fractions showed the presence of a DNA peak in high density fractions (heavy DNA) from T1 and T2 samples from all treatments in addition to the main (unlabeled) DNA peak present (**Supplementary Figure S1**).

**FIGURE 6:**
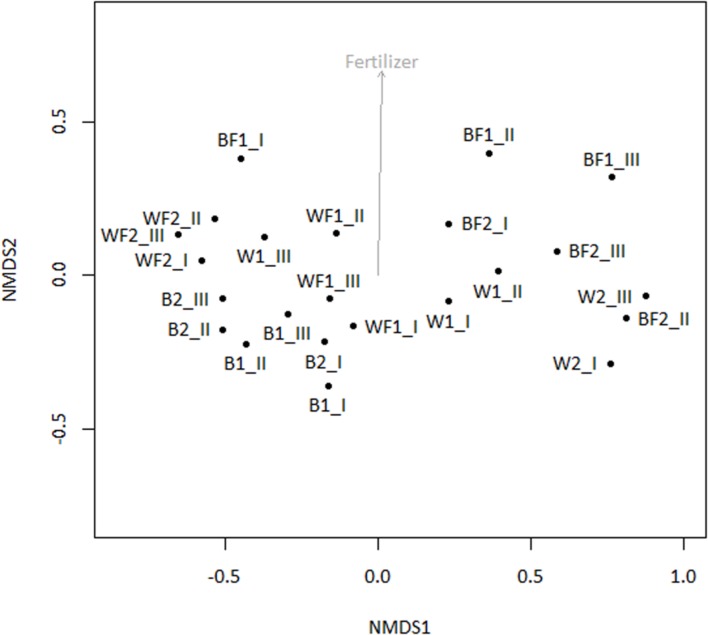
**NMDS of 16S rRNA genes amplified from ^13^C-enriched DNA with subsequent fitting of environmental vectors of treatment (willow, fertilizer, time) onto the ordination (*P* ≤ 0.05)**.

Pyrosequencing of 16S rRNA genes amplified from ^13^C-enriched DNA followed by NMDS (**Figure 6**) indicated that fertilizer was a significant variable (vector fitting, *P* ≤ 0.05) that could explain the ordination. These findings echoed the rates of evolved ^13^CO_2_, which were significantly increased in fertilized treatments. PERMANOVA analysis further demonstrated that there was an interactive effect of willow and fertilizer on the ^13^C-labeled microbial community (*F* = 14.49, *p* = 0.01), but no individual effects of either willow or fertilizer (*P* > 0.1). The major taxa involved in the acquisition of carbon from naphthalene are shown in **Table 1**. In most libraries, *Pseudomonas* and *Acidovorax* genera had the highest relative abundance. In fertilized bulk soil, *Elizabethkingia* was high in abundance, whereas in willow-vegetated soil and fertilized bulk soil, *Brevundimonas* was high in abundance. Other, less dominant populations, belonged mostly to *Proteobacteria* with most of the diversity being represented by *Burkholderiales*, including the genera *Acidovorax*, *Variovorax*, *Hydrogenophaga*, *Polaromonas*, *Achromobacter/Bordetella*, or as yet unclassified genus of the *Comamonadaceae*.

**Table 1:** Taxa deriving carbon from naphthalene, either directly or via cross-feeding, as detected at different time points.

Taxon (OTU)*^a^*	Closest RDP SeqMatch type strain of the OTU representative sequence	GenBank accession no.	Similarity score*^b^*	Mean percent of valid reads per treatment*^c^*							
				
				B1	B2	BF1	BF2	W1	W2	WF1	WF2
***Alphaproteobacteria***											
*Caulobacterales*											
*Brevundimonas*	*Brevundimonas vesicularis* LMG 2350	AJ227780	0.996	0.0	0.0	1.7	11.7	1.2	40.7	0.0	0.0
*Rhizobiales*											
*Rhizobium*	*Rhizobium radiobacter* IAM 12048	AB247615	0.990	0.0	0.0	0.0	1.1	0.0	0.9	0.0	0.0
***Betaproteobacteria***											
*Rhodocyclales*											
*Georgfuchsia*	*Georgfuchsia toluolica* G5G6	EF219370	0.930	1.5	1.7	0.2	0.0	0.1	0.0	0.1	0.0
*Burkholderiales*											
*Acidovorax*	*Acidovorax defluvii* BSB411	Y18616	0.998	19.7	24.1	21.9	36.9	53.3	1.8	37.0	12.7
*Variovorax*	*Variovorax paradoxus* DSM 66	AJ420329	0.994	0.0	0.1	0.5	1.1	0.0	0.0	0.0	0.0
*Hydrogenophaga*	*Hydrogenophaga taeniospiralis* ATCC 49743	AF078768	0.994	1.1	1.6	5.1	0.6	0.9	0.0	1.4	10.1
*Polaromonas*	*Polaromonas jejuensis* JS12-13	EU030285	0.998	0.4	0.5	0.3	1.1	1.3	0.0	0.1	0.1
*Achromobacter/Bordetella*	*Achromobacter xylosoxidans* DSM 10346	Y14908	0.996	0.0	0.0	3.2	0.0	1.9	0.0	0.0	0.0
unclas. *Comamonadaceae*	*Rhodoferax antarcticus* ANT.BR	GU233447	0.986	2.1	2.8	0.8	1.1	0.8	0.0	1.1	1.2
*Methylophilales*											
*Methylophilus*	*Methylotenera mobilis* JLW8	DQ287786	0.994	0.5	0.7	1.0	0.0	0.1	0.0	0.1	0.4
***Gammaproteobacteria***											
*Pseudomonadales*											
*Pseudomonas*	*Pseudomonas* spp.	multiple hits	0.998	68.9	57.0	10.8	7.3	35.8	44.3	56.1	64.2
*Rhizobacter*	*Rhizobacter fulvus* Gsoil 322	AB245356	0.998	0.8	1.1	0.3	0.0	0.1	0.0	0.0	0.2
Unclassified	*Marinobacter lutaoensis* T5054	AF288157	0.940	0.5	0.9	0.0	0.0	0.5	0.0	0.1	0.5
***Deltaproteobacteria***											
*Bdellovibrionales*											
*Bdellovibrio*	*Bdellovibrio bacteriovorus* HD 100	AJ292759	0.977	2.7	5.7	0.0	0.6	0.1	0.0	0.0	0.6
***Bacteroidetes***											
*Flavobacteriales*											
*Elizabethkingia*	*Elizabethkingia miricola* GTC862	AB071953	0.984	0.0	0.0	35.8	27.4	0.0	0.0	0.0	0.0
*Sphingobacteriales*											
*Pedobacter*	*Pedobacter koreensis* WPCB189	DQ092871	0.986	0.0	0.0	0.5	1.1	0.0	0.0	0.0	0.0
***Actinobacteria***											
*Actinomycetales*											
*Arthrobacter*	*Arthrobacter equi* IMMIB L-1606	FN673551	0.996	0.0	0.3	0.0	0.0	0.3	0.0	1.0	2.0


*Only those taxa are shown whose mean abundance was ≥0.5% in at least two libraries. The gray colors indicate higher relative abundance of a taxon in the library. *^a^*Phylogenetic affiliations are based on Ribosomal Database Project (RDP) Classifier. *^b^*Similarity reports the percent sequence identity over all pairwise comparable positions. *^c^*The mean number was calculated from triplicate samples.*

## Discussion

A goal of phytoremediation is to use plants and associated bacteria to extract, stabilize, or degrade xenobiotic pollutants. Although the general concepts of phytoremediation and rhizoremediation are well known (Schnoor et al., 1995; Salt et al., 1998; Siciliano and Germida, 1998; Macek et al., 2000; Kuiper et al., 2004; Macková et al., 2006; Kurzawová et al., 2012), specific mechanisms underlying these technologies are often unclear. This study sought to determine how planting with willow and/or fertilization would affect the microbial community present and active in the transformation of diesel contaminants.

After 12 weeks of growth in contaminated soils, we observed that soils that had been planted with willow had less DRO than soil that was only fertilized or did not have any treatment (**Figure 1**). However, soils that were both planted with willow and fertilized did not produce a significant reduction in DRO after incubation when compared to the unfertilized willow treatment. Under nutrient-rich conditions, willows may produce lower amounts of salicin, which may act to induce microbial biodegradation pathways (Singer et al., 2003; Hartmann et al., 2009), than willows under minimal nutrient conditions, which may account for the decreased degradation of DRO in soils that were both fertilized and planted (Julkunen-Tiitto, 1985). Fertilization also had the effect of delaying willow leaf abscission, which may indicate that root turnover was also delayed. This postponement of fall turnover in the presence of fertilizer may have had an effect on the root lysis and subsequent release of salicin into the rhizosphere (Leigh et al., 2002).

To identify both the culturable and non-culturable bacterial populations potentially involved in degradation of PAHs, SIP with ^13^C-labeled naphthalene was conducted. Aromatics can account for up to 30% of diesel fuel mixtures, and unlike aliphatic hydrocarbons are often more persistent. Therefore, we identified bacterial populations potentially involved in degradation of aromatics through SIP with ^13^C-labeled naphthalene, the simplest PAH. Bacteria deriving carbon from naphthalene may represent a significant portion of the microbial community engaged in degrading PAHs, which constitute some of the more toxic components of diesel fuel. The SIP analysis indicated that the use of willow and fertilizer had a subtle but significant effect on shifting the microbial community structure, and an interactive effect of willow and fertilizer on the bacterial community proved to cause a shift in the identity and activity of bacteria involved in the degradation of naphthalene (**Table 1**, **Figure 6**). Changes in the total bacterial community as measured by 16S rRNA genes were associated with the presence of willow (**Figure 3**).

In the SIP experiment, bacterial communities dominating the acquisition of naphthalene-derived carbon were different depending on treatment. Highly represented genera included *Brevundimonas*, *Rhizobium*, *Acidovorax*, *Pseudomonas*, and *Elizabethkingia* (**Table 1**). In untreated soils and both soils impacted by willows, *Pseudomonas* predominated the labeled community, similarly to many previous reports (Jones et al., 2011; Lu et al., 2011; Wald et al., 2015). Members of the genus *Pseudomonas*, such as *P. putida*, *P. stutzeri*, and others, are common rhizosphere bacteria whose mechanisms of and factors affecting naphthalene degradation have been thoroughly investigated (Dunn and Gunsalus, 1973; Rosselló-Mora et al., 1994). The classic NAH7 catabolic plasmid containing naphthalene degradation genes in two operons was first described in *P. putida* G7 (Dunn and Gunsalus, 1973). Many different catabolic plasmids have since been discovered in pseudomonads, and several properties of pseudomonads have been described that enhance their ability to degrade naphthalene such as redundant dioxygenases necessary for PAH degradation (Harayama and Timmis, 1989; Abbasian et al., 2015).

In unfertilized willow soils, most abundant members of the metabolically active community changed over time, with *Acidovorax* dominating after 3 days of incubation, and *Brevundimonas* representing the majority of the community after 7 days. *Acidovorax* species are also commonly associated with PAH-contaminated sites and are more well-known for degrading phenanthrene, a three-ringed PAH, but are also capable of degrading naphthalene and other PAHs (Singleton et al., 2009). *Brevundimonas vesicularis* has also previously been implicated in the oxidation of naphthalene and phenol (Juhasz and Naidu, 2000; Whiteley et al., 2001).

In soils that were fertilized but unplanted, *Elizabethkingia* and *Acidovorax* were the two most dominant genera to derive carbon from naphthalene across both time points, with *Elizabethkingia* being higher in abundance after 3 days and *Acidovorax* increasing in abundance after 7 days. *Elizabethkingia* is a member of the order *Flavobacteriales* which has been previously implicated in hydrocarbon degradation (Khan et al., 2013). However, to our knowledge, this is the first instance of *Elizabethkingia* being implicated in the processing of carbon derived from a PAH.

It has long been known that vegetation and fertilizer can cause shifts in the bacterial community present in soils (Benizri and Amiaud, 2005; Liliensiek et al., 2012; Leff et al., 2015; Su et al., 2015). We hypothesized that willow would have a direct influence on the total community and on the community involved directly in processing carbon from naphthalene. Our data partially support this hypothesis. In the pot study, willow had a significant role in structuring the bacterial community (**Figure 3**) and is associated with significant decreases in DRO (**Figure 1**). However, the SIP study indicated that the effects of fertilizer were driving the differences seen in community function as measured by substrate mineralization (**Figure 5**) and in community structure (**Figure 6**). Finally, analysis of the total variance in both the pot study and SIP study indicated an interactive effect between willow and fertilizer on the bacterial communities. Willow growth in the absence of fertilizer resulted in lower overall plant biomass and earlier leaf senescence, potentially indicating that the fertilized plants had increased fitness and therefore higher rates of survival across seasons. This study indicates that fertilizer has a dual role in phytoremediation: both by promoting the establishment and health of willow trees and by inducing microbial populations to process contaminants at increased initial rates. After initial fertilization, microbial metabolism of contaminants could be expected to continue in the established willow community across the long term, as was shown previously in a long-term field study (Leewis et al., 2013).

Determining how a plant’s presence impacts the identity and diversity of naphthalene-degrading soil communities is important for choosing an appropriate plant for phytoremediation. While many plants stimulate naphthalene removal from the soil, not all do, and some may even inhibit removal compared to bulk soil (Siciliano et al., 2003). It is therefore important to consider specific plant-microbe interactions when optimizing and implementing phytoremediation treatments. Willows are well-suited for use in phytoremediation as they colonize disturbed areas, are fast growing, and naturally occur throughout a wide range of habitats, including the Arctic and sub-Arctic (Johnson et al., 2009). The use of native plants has gained increased attention (Slater et al., 2011; Leewis et al., 2013) due to the increasing threat of invasive species and their nascent ability to thrive in harsh environments. This study clearly demonstrates that willows native to Alaska and north-western Canada function by accelerating DRO degradation, and with fertilizer, increase aromatic degradation by certain indigenous bacterial taxa.

## Author Contributions

MCL and OU wrote the manuscript. KM, SF, AK, CG, and OU conducted the experiments. MCL and OU analyzed the data. MBL and KM designed the experiment. MBL, TM, and OU provided the equipment and supplies.

## Conflict of Interest Statement

The authors declare that the research was conducted in the absence of any commercial or financial relationships that could be construed as a potential conflict of interest.

## Supplementary Material

**FIGURE S1 | Distribution of DNA throughout the gradient fractions.** T0, T1, T2 represent the sampling times of day 0, day 3, and day 7, respectively.
